# Nephrotoxicity Associated with Novel Anticancer Agents (Aflibercept, Dasatinib, Nivolumab): Case Series and Nephrological Considerations

**DOI:** 10.3390/ijms21144878

**Published:** 2020-07-10

**Authors:** Luca Piscitani, Vittorio Sirolli, Lorenzo Di Liberato, Manrico Morroni, Mario Bonomini

**Affiliations:** 1 Nephrology and Dialysis Unit, Department of Medicine, G. d’Annunzio University, Chieti-Pescara, SS. Annunziata Hospital, Via dei Vestini, 66013 Chieti, Italy; lucpis90@virgilio.it (L.P.); vittorio.sirolli@unich.it (V.S.); lorenzo.diliberato@asl2abruzzo.it (L.D.L.); 2 Department of Experimental and Clinical Medicine-Neuroscience and Cell Biology, School of Medicine, Università Politecnica delle Marche, Via Tronto 10/A, 60126 Ancona, Italy; m.morroni@univpm.it

**Keywords:** nephrotoxicity, cancer, kidney, VEGF, anti-VEGF agents, immune checkpoint inhibitor, aflibercept, dasatinib, nivolumab

## Abstract

Cancer patients have an incidence of about 60% kidney disease development and are at elevated risk of acute renal damage. Kidney disease in these patients is frequently associated with nephrotoxicity from the ongoing oncological treatment. New anticancer therapeutic strategies, such as targeted therapies and immunotherapies, offer substantial benefits in the treatment of many neoplasms. However, their use is associated with significant nephrotoxicity, which qualitatively differs from that seen with traditional cytotoxic chemotherapy, while the underlying mechanisms are complex and still to be clearly defined. Nephrologists need to be knowledgeable about the array of such renal toxicities for effective collaboration with the oncologist in the prevention and management of kidney involvement. Renal adverse effects may range from asymptomatic proteinuria to renal failure, and their prompt identification and timely treatment is essential for optimal and safe care of the patient. In this article, after presenting clinical cases we discuss the differing renal toxicity of three novel anticancer agents (aflibercept, dasatinib, and nivolumab) and possible measures to counter it.

## 1. Introduction

Onco-nephrology is today an established, evolving subspecialty that encompasses the complex relationship between cancer and the kidneys. On the one hand, the presence of kidney disease, particularly in the setting of reduced renal function, needs to be taken into consideration in cancer therapy management, since it jeopardizes continuing potentially curative chemotherapeutic regimens [1]. On the other hand, cancer may cause kidney damage through several direct or indirect mechanisms, including the involvement of renal parenchyma, volume depletion, tumor lysis syndrome, hypercalcemia, and myeloma kidney. It must be noted, also, that the number of nephrotoxic oncology medications has significantly increased over the last few decades [2].

The emergence of effective and well-tolerated therapies targeting specific genes/proteins and novel immunotherapies, while allowing chemotherapy-refractory malignancy to be treated, has however increased the occurrence of kidney-related adverse events [2,3]. Clinical manifestations may include acute kidney injury, glomerular injury with proteinuria, electrolyte disturbances, hypertension, tubular dysfunction, and at times chronic kidney disease [4]. The nephrotoxicity profiles of these therapies are qualitatively different from those observed with conventional cytotoxic chemotherapy [4]. Kidney injury may derive from several differential mechanisms, depending not only on the class but also on the single agent [5]. Thus, the practicing nephrologist has to become familiar and develop expertise with the potential nephrotoxicity of new anticancer medications, particularly their associated clinical and laboratory manifestations, given their wide use and the increased number of patients surviving cancer.

We report here three cases which display multiple different aspects of the nephrologist’s involvement in differential renal toxicities from three commonly used new anticancer agents (aflibercept, dasatinib, and nivolumab), and discuss them in the context of the most recent literature, focusing not only on treatment but also on the prevention/minimization of renal damage.

## 2. Case Reports

### 2.1. Case 1

**Figure 1:**
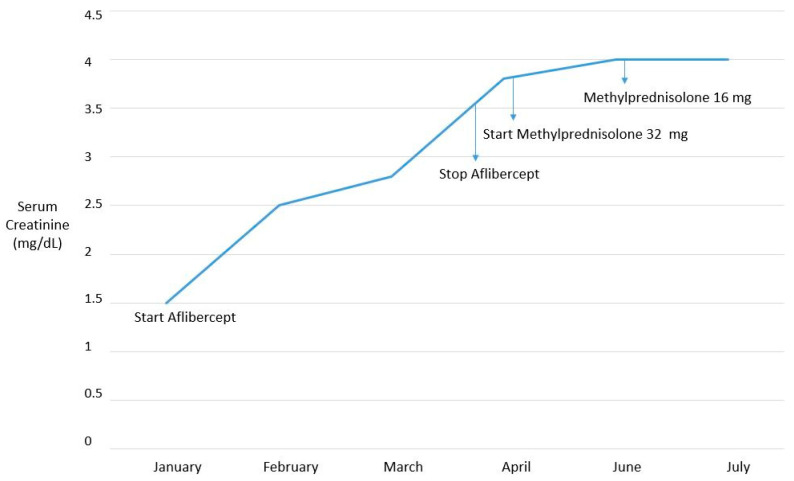
Time course of renal function over time.

A 54-year-old male patient with a history of high blood pressure was efficaciously treated with lisinopril 20 mg/day. A positive fecal occult blood screening had raised the suspicion of a neoplastic pathology of the rectum, which was confirmed by a subsequent colonoscopy. An abdomen computerized tomography (CT) with contrast medium, carried out for staging of the disease, showed multiple hepatic metastases and a left kidney wrinkle. Biochemical tests showed slightly reduced renal function, with serum creatinine at 1.3 mg/dL (estimated Glomerular Filtration Rate, eGFR, 61.9 mL/min by Chronic Kidney Disease—Epidemiology Collaboration, CKD-EPI, equation). The patient underwent a left hemicolectomy and the resection of liver metastases. An abdomen CT scan one year after surgery showed the recurrence of the disease in the liver, and the patient started chemotherapy with oxaliplatin, 5 fluorouracil, and cetuximab every two weeks for six months. On January 2019, the therapeutic regimen was modified to include aflibercept (anti-Vascular Endothelial Growth Factor, VEGF), 5 fluorouracil (pyrimidine analogue), and irinotecan DNA polymerase inhibitor. At that time, the serum creatinine was 1.5 mg/dL (eGFR 52 mL/min). After three months of therapy, with a cumulative dose of 20 mg/kg aflibercet there was severe worsening of hypertension no longer controlled by lisinopril, which was associated with the gradual and progressive worsening of renal function: the creatinine was 3 mg/dL, with the eGFR at 22 mL/min. The daily urine protein loss was found to be in the nephrotic range (11 g/24 h). There were no direct or indirect signs of hemolysis and the platelet count was normal. Antineoplastic therapy was discontinued. Anti-hypertensive therapy was switched to nifedipine at 30 mg twice daily and furosemide at 25 mg/die. After 15 days, as the further worsening of renal function was found (creatinine at 3.8 mg/dL), steroid therapy (methylprednisolone at 32 mg/day) was started. Anti-hypertensive therapy was upgraded by including doxazosin 4 mg × 2/die and increasing furosemide to 25 mg twice a day. Since 24 h blood pressure monitoring showed the mean of systolic and diastolic blood pressure to be 170 and 110 mmHg, respectively, minoxidil at 5 mg twice a day was added, obtaining an improved control of blood pressure (140/90 mmHg). After two months, the serum creatinine was 4 mg/dL and the methylprednisolone was tapered to 16 mg/day; the proteinuria decreased to 3.5 mg/dL. One month later, the renal function proved to have plateaued (Figure 1). The patient then moved to another city and was lost to follow-up. He died of cancer three months later.

### 2.2. Case 2

**Figure 2:**
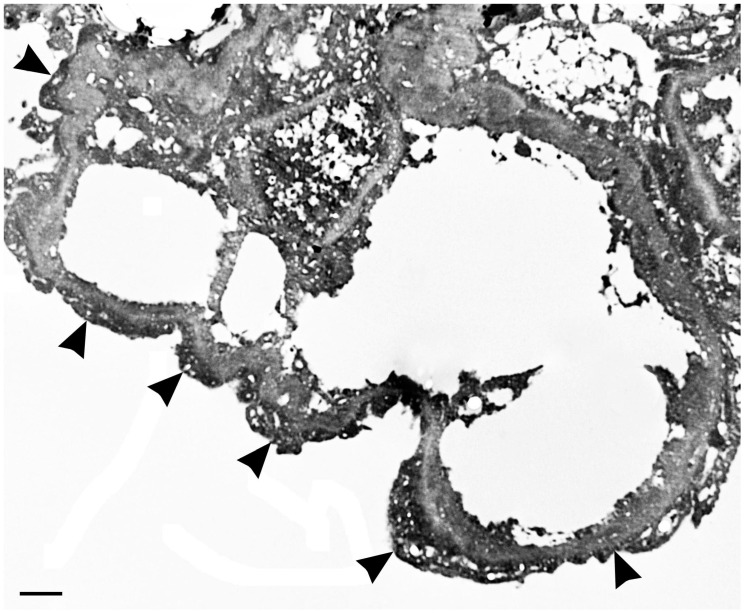
Electron micrograph revealing diffuse foot process effacement over the entire capillary surface (arrowheads).

Case two refers to a 43-year-old woman who was in good health until March 2014 when, in the absence of clinical symptoms, a routine blood count examination documented an increase in myeloid line cells associated with lymphocyte reduction at a peripheral smear. An osteomedullary biopsy was found to be diagnostic for chronic myeloid leukemia. Therapy with hydroxyurea and allopurinol was initiated. In April 2014, following a positive finding for the Philadelphia chromosome, the ongoing therapy was suspended and treatment with imatinib was begun. It was maintained until February 2016, when, due to the onset of nausea refractory to symptomatic agents, the therapy was switched to dasatinib at 100 mg once a day. Laboratory tests documented normal renal function. In September 2017, for the first time the patient performed a urine stick that revealed proteinuria at 200 mg/dL. The daily urinary protein loss was 7.630 g, and the patient was referred to our unit. During hospitalization, laboratory tests confirmed proteinuria with normal renal function (serum creatinine at 0.9 mg/dL). A renal biopsy documented the presence of minimal-change glomerulonephritis. The glomeruli in hematoxylin-eosin were normal except for minimal basement membrane thickening. Complement and immunoglobulins were absent on immunofluorescence. Electron microscopy revealed diffuse foot process effacement over the entire capillary surface (Figure 2).

It was recommended that the dasatinib therapy be discontinued. Regular nephrological checks documented the progressive reduction in proteinuria. In February 2019, proteinuria was absent and the renal function remained normal (serum creatinine at 0.9 mg/mL). The absence of proteinuria and normal renal function were confirmed in February 2020. The oncologist did not resume any antineoplastic agent.

### 2.3. Case 3

**Figure 3:**
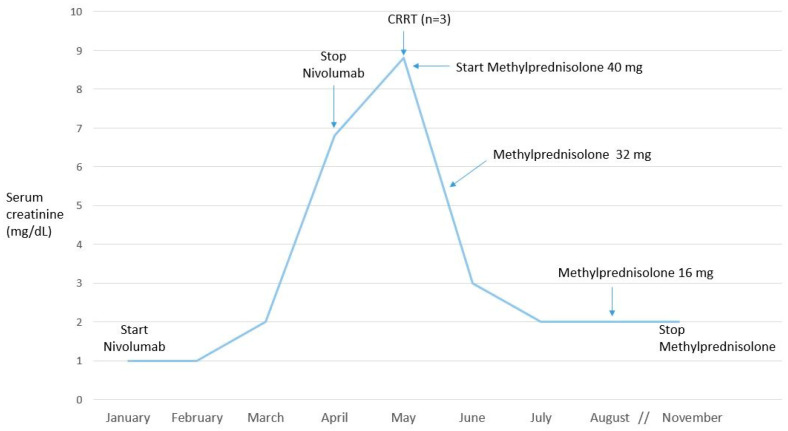
Time course of renal function over time.

Case 3 refers to a 58-year-old patient who had had hypertension for about 20 years. In 2016, the patient underwent left lower pulmonary lobectomy due to adenocarcinoma. After about a year, an abdominal CT scan documented a neoformation in the left kidney which required enucleation (clear-cell renal carcinoma at histology). At a subsequent chest CT, multiple bilateral, non-calcific nodular pulmonary lesions were documented, for which atypical sub-segmentary pulmonary resection surgery was performed (including a nodular lesion of the lower left lobe) by means of video-assisted mini-toracotomy; a histological examination was diagnostic for a metastatic lesion of adenocarcinoma with pulmonary primitiveness without EGFR, ELK, or ROS 1 mutation. In February 2017, cisplatin and pemetrexed chemotherapy was initiated, administered at 3-week intervals for a total of four cycles. Monotherapy with pemetrexed was continued until October 2017, when the oncologist suspended chemotherapy for three months due to abundant lacrimation. Laboratory tests documented the normality of both renal function and urinalysis. In December 2017, Nivolumab therapy was started at 240 mg every two weeks. In March 2018, for the first time the renal function proved to be altered (serum creatinine at 2 mg/dL, eGFR at 66.3 mL/min) in the absence of proteinuria or microhematuria in the urine test. The renal function over the following weeks showed a rapid deterioration, with creatinine at 6.5 mg/dL; diuresis was preserved (as was observed throughout the hospital stay). In light of this clinical picture, and in agreement with the oncology specialist, therapy with nivolumab was suspended. Despite the suspension, however, the renal function worsened further, with creatinine up to 8.8 mg/dL (Figure 3). The patient was then admitted to our unit where, the severe worsening of renal function being confirmed, a jugular central venous catheter was placed and hemodialysis (CRRT) was initiated for a total of three sessions. Steroid therapy with methylprednisolone at 40 mg/day i.v. was also introduced and maintained during hospitalization, then replaced by oral methylprednisolone at 32 mg/day. A renal biopsy could not be performed due to previous enucleation in the left kidney. A progressive improvement in renal function was observed, and at discharge the creatinine was 3 mg/dL. An oral methylprednisolone 32 mg/day regimen was maintained for 3 months and then gradually tapered off. During two years’ follow-up, the patient’s renal function proved to be stabilized (serum creatinine at 2 mg/dL), without the need for dialysis therapy.

## 3. Discussion

The following discussion divides into different sections, covering the nephrotoxicity of the different classes of pharmacological agent described in our case reports, followed by some nephrological considerations concerning attempts to combat renal damage.

### 3.1. Vascular Endothelial Growth Factor Signaling Pathway Inhibitors

In 1971, angiogenesis was proposed for the first time by Folkman as a mechanism for growth and tumor metastases [6]. VEGF is the main promoter of angiogenesis [7]; it induces endothelial fenestrations and modulates vascular permeability. VEGF is expressed in all tissues during development and in vascular tissues in adults. In human renal tissue, VEGFA is the most abundant isoform of VEGF. VEGFA acts by binding to one of two receptors—VEGF receptor 1 (VEGFR1) and VEGFR2 [8]—and downstream signaling pathways such as MAPK/ERK 1/2, endothelial nitric oxide synthase, and the anti-mammalian target of rapamycin [5]. Both VEGF receptors are involved in angiogenesis, though VEGFR2 is responsible for most VEGFA signaling [9].VEGFR1 also exists in a soluble form, which acts as a decoy receptor, inhibiting VEGFA signaling [10]. In the kidney, VEGFA is expressed in podocytes and in the tubular epithelium, while its receptors are found in the mesangium and the glomerular and peritubular capillaries [5]. The functions of constitutively expressed VEGFA and VEGF receptors in the kidney are reported in Table 1.

The pharmacologic inhibition of VEGFA/VEGFR signaling and its downstream pathways is a common therapeutic strategy in the oncology setting, where new drugs are continuously being developed. VEGF signaling pathway inhibitors can be classified according to their target of action in the VEGFA-VEGFR2 pathway as drugs inhibiting VEGFA, sequestering VEGFA, inhibiting receptor tyrosine kinases, or inhibiting downstream pathways [5,11].

Renal adverse effects may include arterial hypertension, asymptomatic proteinuria, nephrotic syndrome, and acute renal failure [11]. The mechanisms of renal toxicity in VEGF signaling pathway inhibitors, particularly endothelial and glomerular injury, may vary among the different classes of such agents, and most of them are not clearly understood [5].

#### 3.1.1. Aflibercept

Aflibercept was approved for the treatment of cancer by the Food and Drug Administration (FDA) in 2011. It is a recombinant fusion protein which comprises binding domains for VEGFR1 and VEGFR2 attached to the Fc region of human IgG1 [6], and acts as a VEGF trap for soluble VEGF enzyme [12].

Aflibercept, as well as all other VEGF-targeted therapies, can cause or worsen hypertension, one risk factor being a pre-existing hypertensive state [13]. In a prospective single-center study concerning an observational cohort of patients referred for hypertension, proteinuria, and/or renal failure, all seven patients treated with aflibercept required antihypertensive drugs [14]. In a meta-analysis of 15 trials in colorectal cancer patients treated with aflibercept, a 42.4% incidence of all grade (grade1-4) hypertension and a 17.4% incidence of high-grade hypertension was found [15]. High-grade hypertension was a combination of grade 3 (requirement of therapy or more intensive therapy than previously) and grade 4 (hypertension crisis). The use of aflibercept was associated with a higher risk of developing hypertension than bevacizumab treatment [15].

**Figure 4:**
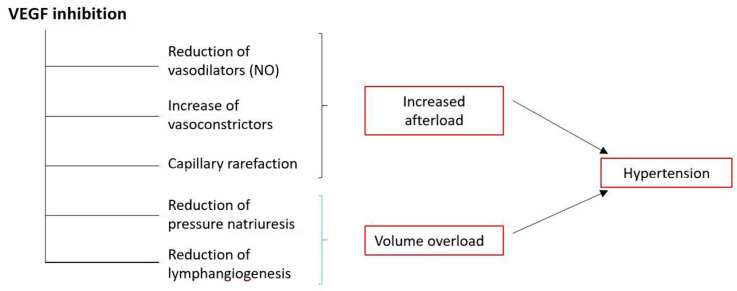
Proposed mechanisms in the genesis of hypertension by targeted anti-VEGF therapy. Modified from 18.

There are several mechanisms whereby anti-VEGF agents including aflibercept may cause hypertension (Figure 4). The inhibition of VEGF reduces the transcription of endothelial nitric oxide synthase and hence the production of nitric oxide [16]. Decreased nitric oxide can increase blood pressure via both vasoconstriction and sodium retention [11,17,18]. In addition, the inhibition of the VEGF pathway could lead to hypertension through a reduction in the density of microvessels (capillary rarefaction), with a consequent increase in peripheral resistance [18]. Finally, increased oxidative stress can also contribute to hypertension [11], VEGF being protective against endothelial damage secondary to oxidative stress [19].

The increased loss of proteins in urine (proteinuria) represents a common event in cancer patients treated with angiogenesis inhibitors, though its incidence may be vary from study to study. Significant predictors for the development of proteinuria may include the number of cycles (>13), the systolic blood pressure at baseline ≥ 135 mmHg, and the use of calcium channel blockers [20]. Systemic hypertension is a postulated pathogenic mechanism in the development of such proteinuria, through an increase in intraglomerular pressure [11]. However, there is increasing evidence based on renal histology data that anti-VEGF therapy can lead to various kidney injuries causing proteinuria [4,5]. The excretion of protein in urine in normally hampered by the glomerular filtration barrier, which is formed of endothelial cells, podocytes, and basement membrane components. VEGF is produced by podocytes and binds to VEGFR-2 expressed by endothelial cells. This interaction is of critical importance for both normal function and the repair of the system [14]. The inhibition of VEGF in podocytes indeed has been shown to result in the loss of endothelial fenestrations in the glomerular capillaries, the proliferation of glomerular endothelial cells, the loss of podocytes, and the urinary excretion of proteins [21].

In the few cases of biopsy-proven renal toxicity by aflibercept, thrombotic microangiopathy (TMA) characterized by luminal thrombosis within the glomerular capillary was consistently found [14,22,23]. It must be noted that aflibercept-associated TMA, unlike other more aggressive iatrogenic TMA [24], seems to be renal-specific (intraglomerular exclusively), with biological disorders such as thrombocytopenia or schistocytosis in only half of the patients [23]. Renal involvement may occur at variable times after the beginning of aflibercept and include hypertension, proteinuria up to the nephrotic range (>3.5 g/die), and/or renal insufficiency (creatinine clearance <60 mL/min per 1.73 m^2^). A decrease in both proteinuria and blood pressure together with the improvement of renal function was found only when VEGF-trap was withdrawn and antihypertensive medications were used [14]. Molecular studies on glomeruli in biopsied kidney samples from patients treated with anti-VEGF agents (bevacizumab or aflibercept) have shown undetectable VEGF expression, a high abundance of RelA (both glomerular endothelial cells and podocytes), and no detection of c-Maf-inducing protein (c-mip) [23]. The upregulation of ReIA, a subunit of the transcription factor nuclear factor-kB (NF-kB), indicates increased NF-kB activity and is proven to directly suppress c-mip activity by binding to its promoter [23]. Since the activation of NF-kB induces proinflammatory cytokines and increases the intrarenal renin angiotensin system caused by proteinuria, blocking it might offset the renal damage associated with persistent proteinuria [25]. The inhibition of NF-kB could therefore be therapeutically useful in treating aflibercept-associated TMA, though this issue remains to be validated in future studies.

Upon the initiation of aflibercept treatment, the patient reported here (case 1) displayed a poor control of blood pressure and proteinuria in the nephrotic range. Despite the discontinuation of VEGF-trap, as suggested by guidelines [26,27], and intensive anti-hypertensive polipharmacotherapy, the renal function continued to worsen. Thus, though no actual histological diagnosis, which may influence treatment options and prognosis [4], was obtained since kidney biopsy was not feasible, corticosteroid therapy was started. After two months, though kidney function did not recover unlike a previous case [22] (possibly due to the slightly reduced renal function of our patient at baseline), stabilization and no further apparent worsening was observed, as confirmed a month later (Figure 1). One can therefore report that corticosteroids may represent a rescue therapy in aflibercept-treated patients with serious renal complications, particularly severe proteinuria or persistent renal insufficiency, although clearly further evidence is needed to support such a strategy.

#### 3.1.2. Dasatinib

Dasatinib is one of the tyrosine kinase inhibitors (TKIs) of VEGF signaling. Several TKIs have been approved for clinical use. They have distinctive pharmacodynamic properties [5], and their effects on the kidney appear to be agent-specific. TKIs interfere with the activity of one or more families of receptor tyrosine kinases, including VEGFR, fibroblast growth factor receptor, platelet-derived growth factor receptor, and epidermal growth factor receptor, which all share a similar structure [5].

Dasatinib is a second-generation TKI which inhibits the activity of breakpoint cluster region-abelson (BCR-ABL)-kinase; kinases from the SRC family; and several other oncogenetic kinases, including PDGF beta receptor, ephrin receptor, and c-kit. It has a short half-life and the ability to act on the activated form of BCR-ABL protein, which distinguishes dasatinib from other TKIs such as imatinib and nilotinib. Dasatinib is approved for the treatment of adult patients suffering from newly diagnosed Philadelphia chromosome-positive (Ph+) chronic myeloid leukemia, Ph+ acute lymphoblastic leukemia, and chronic myeloid leukemia resistant to previous therapy in either the blast, accelerated, or chronic phase.

Though preclinical studies and multiphasic clinical trials failed to identify serious renal toxicity, real-life experience with dasatinib has evidenced some uncommon renal adverse effects. The recent mining of the FDA Adverse Reporting System database has demonstrated that, among several FDA-approved kinase inhibitors, dasatinib is associated with a high risk of nephrotoxicity, particularly proteinuric glomerular disease, with no apparent increase in hypertension risk [28]. Three case reports of acute renal failure have been reported, although none were definitely linked to dasatinib [4].

The incidence of proteinuria in dasatinib-treated patients proved to be 18% in a phase I dose-escalation and pharmacokinetic study, with a much lower incidence of grades 3–4 proteinuria [29]. The severity of proteinuria seems to be dose-dependent [30]. To our knowledge, including the present one there are nine case reports of nephrotic syndrome caused by dasatinib reported in the scientific literature. Three cases affected children [31,32,33], and the remainder adults [30,34,35,36,37]. Nephrotic proteinuria developed after a duration of dasatinib administration ranging from 2 weeks [30] to 2 years [37]. Dose reduction [30] or the discontinuation [31,32,33,37] of dasatinib was associated with an improvement in proteinuria. Switching to a first-generation TKI such as imatinib [35,36] or nilotinib [34], or to the new second-generation TKI bosutinib [37], also proved an effective strategy.

In the case reported here (patient 2), upon the identification of the underlying podocytopathy and taking into consideration the normal renal function, dasatinib was discontinued with approval by the oncologist and no further therapy such as steroids was employed. Nephrological monitoring over time confirmed the disappearance of proteinuria and the maintenance of normal renal function as well.

Biopsy specimens obtained in some cases of dasatinib-associated nephrotic proteinuria [31,32,34,35,37] revealed podocytopathy (minimal change disease), characterized by the effacement of the podocyte foot processes at electron microscopy analysis, as in our patient (Figure 2). Podocytes are crucial for the establishment of the blood-urine filtration barrier in glomeruli, and the injury of them causes proteinuria. Nephrotic-range proteinuria in dasatinib patients has generally been attributed to the disruption of the VEGF signaling pathways through the inhibition of the Src family of kinases [38,39], which in turn inhibits VEGF production [40]. VEGF is produced in podocytes and, as an autocrine effect, binds to the VEGFR-2 of its own podocyte, thereby controlling both the cytoskeleton and slit diaphragm among the podocyte foot processes [41]. It is thus possible that dasatinib-induced glomerular damage could be similar to that occurring with other VEGF inhibitors [38].

However, recent findings indicate that dasatinib nephrotoxicity is independent of systemic or glomerular VEGF inhibition but rather primarily due to its direct effect on the podocyte cells [28].

Dasatinib-treated podocytes show significant changes in the focal adhesion, actin cytoskeleton, and morphology, which are not observed with many other TKIs tested [28]. Quantitative phosphoproteomics identified 76 statistically downregulated proteins, related to the regulation of the actin cytoskeleton and focal adhesion. This unique cytoskeletal phenotype found in podocytes treated with dasatinib as compared to other KIs, and proved to be induced by the inhibition of LIM kinase [28], a key regulator for the formation and crosslinking of actin stress fibers [42]. In addition, the atomic force microscopy elastography technique showed a significant and marked reduction in the mean cellular elasticity of dasatinib-treated podocytes. Furthermore, the chronic administration of dasatinib in mice resulted in the effacement of podocyte foot processes, thereby confirming the in vitro observation of a substantial cytoskeletal effect.

Podocytes have exceptionally fragile cytoskeletal dynamics [43], and the loss of their biomechanical integrity may represent a consistent signature underlying many glomerular disease models [44]. Dasatinib could induce glomerular toxicity through a direct effect on the structural integrity of podocyte cytoskeleton, leading to decreased cellular elasticity impinging on its key function as a structural member of the filtration barrier [28]. Such a unique nephrotoxicity mechanism would distinguish dasatinib from other TKIs.

### 3.2. Immune Checkpoint Inhibitors

Immune checkpoint inhibitors (ICPs) have recently emerged as a frontline treatment for various types of malignancies. Immune checkpoints are regulatory proteins that act to maintain a balance between activating T cells to enhance the destruction of foreign antigens (such as cancer cells) and suppressing T-cell activation against self-antigens [45]. Cancer cells combat the immune system by activating immune checkpoints in order to evade immune surveillance [46,47]. ICPs can target either programmed cell death-1 and ligand (PD-1 and PDL-1, respectively), or cytotoxic T lymphocyte antigen-4 (CTLA-4). The binding of the PD-1 protein on T lymphocytes to PDL-1 expressed on cancer cells deactivates the T cells, thereby protecting the tumor cells from destruction by the immune system. Monoclonal antibodies that interrupt the interaction between PDL-1 and the T cell PD-1 receptor allow activated tumor-infiltrating T cells to destroy cancer cells [48,49]. The monoclonal antibodies blocking PD-1 receptors include nivolumab, pembrolizumab, and cemiplimab, while atezolizumab, avelumab, and durvalumab block PD-L 1 [45]. ICPs are also considered monoclonal antibodies targeting CTL-4, such as ipilimumab and tremelimumab (the latter undergoing evaluation). CTL-4 expressed on the CD4+ T-helper cell surface transmits an inhibitory signal to T cells. The blocking of CTL-4 prevents this signal, thus improving the antineoplastic response [50].

ICPs are not excreted by the liver or the kidneys, have a long half-life, and undergo receptor-mediated clearance. They have proved effective in the treatment of a broad spectrum of cancers [51]. However, their use comes at the cost of autoimmune phenomena known as immune-related adverse events [52]. A variety of organs can be affected, though skin and the gastrointestinal tract seem to be the most commonly involved [53]. Renal complications appear to be less common [53,54], although they are becoming increasingly recognized since the use of these agents continues to expand [55], and there may be an underestimation according to autopsy series [56].

#### Nivolumab

Nivolumab is an immunoglobulin G4 monoclonal antibody that acts by targeting the PD-1 receptor, as previously described. It was approved in 2014 by the FDA for the treatment of subjects with metastatic melanoma [57], and since then this immunotherapeutic approach has been used for several other cancers. Renal toxicity more commonly manifests as acute kidney injury (AKI). An analysis of all published phase II and III clinical trials that included at least 100 patients showed nivolumab-treated patients (*n* = 1489) to have a 1.9% incidence of any AKI and a 0.3% incidence of grade 3 or 4 AKI [54]. AKI occurred more frequently in patients (*n* = 407) who received nivolumab plus ipilimumab (4.9% and 1.7%, respectively).

Renal biopsy data demonstrate that acute tubulointerstitial nephritis (ATIN) is the most common pathological finding in AKI patients treated with nivolumab alone or in combination with other ICPs [54,58,59,60,61,62,63,64]. AKI developed after a variable time course from CPI exposure, ranging from some weeks to several months. A recent report, however, showed AKI occurring within a few days of the first administration of nivolumab [65]. The majority of the patients had sub-nephrotic proteinuria and pyuria, whereas fever, rash, and eosinophilia were absent in most. Either complete or partial renal recovery was obtained with drug discontinuation and steroid therapy [45]. It is noteworthy that the majority of patients developing AKI were also receiving drugs known to be associated with ATIN, mainly proton-pump inhibitors but also non-steroidal anti-inflammatory drugs and antibiotics [45,54,58,64]. This suggests that ICP treatment can lead to the loss of tolerance via the activation or reactivation of drug-specific T cells in some patients [66]. The discontinuation of a potential culprit drug is recommended [45], since its cessation would lead to a more rapid attenuation of immunologic activity [62].

That ATIN is the most common kidney lesion in patients receiving ICPs including nivolumab has been confirmed in a recent multicenter retrospective study [66] focusing on the most clinically significant episodes of ICP-AKI (the doubling of serum creatinine or the need for renal replacement therapy). A lower baseline eGFR, the use of proton pump inhibitors, and combination ICP therapy proved to be each independently associated with an increased risk of AKI. The presence of a concomitant immune-related adverse event was associated with a worse renal prognosis [66].

More recently, newer reports have shown that besides ATIN, nivolumab therapy can cause biopsy-proven glomerular pathologies in association with AKI [64].

Nephrotic syndrome cases due to membranous nephropathy [64,67], focal segmental glomerulosclerosis [64,68,69], and membranoproliferative glomerulonephritis [70] have been reported. Nivolumab discontinuation and steroids were adopted in all patients. This led to complete [64,70] or partial [67] remission, the requirement of additional immunosuppressive medications such as mycophenolate mofetil (because treatment with high-dose corticosteroids had an insufficient effect and as a standard of care to treat the glomerulopathies) to obtain remission though followed by relapse [68], and no recovery in dialysis-dependent end-stage renal disease [69]. IgA nephropathy developed in two patients receiving nivolumab [71,72] and in one patient treated with nivolumab plus ipilimumab [64]. Drug discontinuation [72] plus steroid therapy [64] was associated with remission, whereas the other case showed a more severe AKI and in addition required renal replacement therapies for 5 months before recovery [71]. Acute focal segmental necrotizing pauci-immune glomerulonephritis was also noted in two patients—one treated with nivolumab and one with nivolumab combined with ipilimumab [64]. Both patients completely recovered upon drug discontinuation, the use of steroids, and one dose of rituximab [64].

The cause(s) of nivolumab nephrotoxicity are not clear. Nivolumab-related ATIN could be due to the blockade of PD-1 signaling pathways altering T-cell immune tolerance against kidney intrinsic antigens (autoimmune related) or concomitant drugs (drug-induced) [62]. PD-1/PD-L1 signals play an important role in maintaining peripheral T-cell immune tolerance [73]. The expression of PD-L1 on renal tubular cells protects these cells from T-cell-mediated autoimmunity [74]. It has also been shown that there are some auto-reactive T-cells, which are normally kept dormant by several mechanisms to prevent autoimmunity [75]. It has been proposed that the re-activation of these dormant T-cells by anti-PD1 therapy could disrupt the peripheral immune tolerance between them and renal tubular cells, leading to ATIN [60]. The variety of nivolumab-induced renal manifestations, however, suggests multiple complex mechanisms that remain to be elucidated [64].

Our clinical case reported here (patient 3) indicates ATIN as the possible cause of AKI. The patient developed renal insufficiency after nivolumab therapy, without the occurrence of proteinuria or hematuria, and was not treated with other drug-inducing ATIN. He required renal replacement therapy, which is not common in ICP-AKI [66], in addition to nivolumab discontinuation and steroid therapy (Figure 3). The renal function did not recover completely, but proved to be stable over time. In our case, renal biopsy was not performed due to the previous enucleation of the left kidney.

The need for renal biopsy in the setting of ICP-associated nephrotoxicity is debated [76]. Arguments against [77] are based on the evidence that most AKI cases can be correctly assumed to be caused by ATIN, and that empirical steroid therapy will result in complete or incomplete recovery in most patients without the risk of kidney biopsy complications, which is marked for patients with a single kidney and for those with coagulopathy or thrombocytopenia. Arguments in favor of kidney biopsy [78] are based on the fact that the knowledge of renal lesions can prevent exposing patients to unnecessary steroids (and their possible complications) and potentially enable ICP therapy to be continued in nonimmune-mediated lesions and acute tubular injury. Urinary cytokine biomarkers interleukin-9 and tumor necrosis factor-alpha [79] might in future represent a noninvasive clinical test to identify ATIN.

Finally, there has been a recently reported case of a patient taking nivolumab who was infected by coronavirus infection 2019 [80]. Despite old age and several comorbidities, she had a good clinical course without pneumonia.

## 4. Nephrological Considerations

Nephrotoxicity from targeted therapies and immunotherapies is increasingly being reported. However, not all patients exposed to these agents develop kidney injury, which suggests the presence of factors that could increase the patient’s risk of nephrotoxicity; whenever possible, these should be corrected prior to starting treatment. The identification of high-risk individuals before drug exposure is necessary to prevent or reduce the development of kidney impairment, possibly leading to dose reduction or treatment interruption [81].

Patient characteristics predisposing one to drug-induced nephrotoxicity include older age and female sex, concomitant comorbidities (diabetes, hypertension), hypoalbuminemia, intravascular volume depletion either true (diuretics, vomiting, diarrhea) or effective (congestive heart failure, cirrhosis), and pre-existing renal impairment [38,82]. It is also important to evaluate ongoing and previous therapies, since agents such as nonsteroidal anti-inflammatory drugs and proton pump inhibitors, which are commonly employed in patients with cancer, are known to possibly cause clinical renal syndromes. Renal handling and the innate toxicity of the medication should also be taken into account.

Nephrological workup in a neoplastic patient both before and during anticancer therapy should include the assessment of blood pressure, renal function, and urinalysis. High blood pressure is a particular concern in patients to be treated with anti-VEGF therapies given either systematically or intravitreally [83]. It is recommended that blood pressure should be carefully monitored after starting antiangiogenic treatment for 3–4 weeks and then every 2–3 weeks, with a goal of < 140/90 mmHg [26]. Ambulatory blood pressure monitoring may be necessary in some patients. Although there are no specific data as yet regarding which anti-hypertensive drug should be administered, agents blocking the renin-angiotensin-aldosterone system could be preferred due to their added benefit of decreasing proteinuria. Other anti-hypertensive agents may be needed when the blood pressure remains above the goal. Nitrates (isosorbide mononitrate and isosorbide dinitrate) could be used in cases of reduced NO signaling under VEGF blockade [84].

The measurement of renal function is fundamental for profiling the risk and appropriate dose of any antineoplastic agent in order to guarantee the best cancer treatment possible. How to measure renal function in cancer patients is debated [85]. The direct measurement of GFR is cumbersome. In clinical practice, GFR is estimated by serum creatinine-based formulae, and the CKD-EPI equation appears to be suitable for application in the cancer population [1]. A study analyzing data from 2582 cancer patients examined all the main GFR estimating equations, using a chromium-51-ethylenediaminetetraacetic acid (51Cr-EDTA) GFR measurement as the gold standard [86]. The body surface area-adjusted CKD-EPI method was found to be the most accurate and least biased method published for estimating the GFR. The authors also developed a new model that further improves the estimation of GFR and allows the calculation of predictive confidence intervals for this estimation [87]. If validated in large further databases, this new model may come to be a new standard of care for cancer patients.

A screening urine analysis with the examination of urinary sediment should also be included in the clinical evaluation of the neoplastic patient prior to anticancer therapy. If proteinuria of grade + 1 is present on the dipstick, then the urinary protein excretion should be quantified using 24 h of urine collection or the spot urine protein-to-creatinine ratio (or albumin-to-creatinine ratio).

The simple surveillance described above should be performed regularly when a patient is receiving anticancer treatment, and may enable a prompt recognition of kidney injury. The regular monitoring of renal function should also continue after drug discontinuation, since kidney toxicities may be irreversible even following the discontinuation of treatment, as recently reported with TKIs [88].

Kidney biopsy is the gold standard for revealing diagnostic and prognostic histological features of renal diseases. Since the types of nephropathy even when induced by simple agents, as mentioned in previous sections, may vary enormously, and given the lack of specific clinical and laboratory features, kidney biopsy is indicated in patients with nephrotic proteinuria, evidence of progressive renal disease, unexplained AKI, or nephrotic syndrome. The identification of the glomerular pathology is relevant, because treatment may require other immunosuppressive medications in cases refractory to steroids and as a standard of care in treating glomerulopathies [57]. Considerations for kidney biopsy should always include whether management would be affected, or the risks and benefits in a patient with advanced cancer and possibly limited life expectancy.

To improve the management of nephrotoxicity, we urgently need to develop accurate biomarkers for use in clinical practice. In current times, biomarkers of kidney damage, such as changes in serum creatinine and eGFR and/or urinary albumin/protein excretion, apply to a later stage of kidney injury after substantial kidney injury has occurred. Thus, there is the unmet need for new biomarkers enabling the timely and more accurate detection of kidney dysfunction, which may lead to an improvement in both short- and long-term outcomes [89]. Several cell-based and biomarker-based assays for predicting nephrotoxicity have been developed, and work is underway to examine whether these biomarkers prove useful in clinical drug trials and can be translated to clinical practice [82]. Moreover, the analysis of urine peptide content (urinary peptidomics) may bring a significant improvement in the management of kidney diseases by supporting earlier and more accurate detection, prognostic assessment, and the prediction of treatment response [89]. Recent technical advances as well as certain current and projected ventures hold the promise of clinching the use of urinary peptidomics in the near future as a major part of kidney disease management.

The present study and review originated from the retrospective analysis of some patients who were hospitalized in our Nephrology unit, and focuses on three novel anticancer agents only. This is certainly a limitation. However, these novel agents are widely used in cancer treatment due to the significant improvement they produce in survival and overall prognosis in many neoplasms, and there is increasing recognition of the renal adverse effects that patients can experience. This suggests that particular attention should be paid to possible nephrotoxicity, and close surveillance is required. We recognize here that targeted therapies can have an impact on renal function in multiple ways. We believe that our updated article on the renal toxicities of aflibercept, dasatinib, and nivolumab provide data of interest to both clinicians and researchers.

In all, at the current time there are no established criteria for monitoring patients for renal adverse effects, which can be conducted on a case-by-case basis. Additionally, poor understanding of the pathophysiology associated with renal adverse effects limits the ability to predict and treat nephrotoxicity. Thus, management still largely proceeds by trial and error. Sharing of data (and possibly biological samples) among the scientific community may lead to advancements in the care of cancer patients suffering from nephrotoxicity. An international database register for targeted therapies and their renal adverse effects should also be realized, as suggested [38]. This is an important issue, especially considering that onco-nephrology is an evolving area requiring continuous update as new drugs become available in clinical practice and renal toxicity is observed.

## 5. Conclusions

Despite significant improvements in patient outcome and survival, nephrotoxicity remains a frequent and important complication of anticancer drugs. Renal adverse effects including hypertension, proteinuria, and the worsening of renal function associated with novel targeted therapies and immunotherapies are increasingly recognized in clinical practice, but strategies to mitigate them have not been firmly established [5]. Useful measures, however, include searching for underlying host risk factors, appropriate drug dosing, the avoidance of dehydration, the discontinuation of further potentially nephrotoxic drugs, the adequate control of blood pressure, and the regular monitoring of renal function and urinalysis.

Multidisciplinary management, ideally by a dedicated team, seems to be the key to providing cutting-edge care for patients suffering from cancer and kidney impairment [81]. To be fully integrated in the multidisciplinary team, nephrologists need to be acquainted with these emerging issues [1]. Collaboration with the nephrologist may be needed for renal check-up before any treatment to identify patients who may be at risk of kidney complications, for recognizing and managing the renal side effects of ongoing cancer therapies, for performing a kidney biopsy whenever indicated and feasible and evaluating its result, for treating severe acute cases which may require renal replacement therapy, and for long-term follow-up in surviving patients.

The timely recognition of renal adverse effects connected with targeted therapies such as aflibercept and dasatinib, or with immunotherapies (nivolumab), can aid in the proper management of the cancer patient. A better understanding of the molecular mechanisms whereby these anticancer agents induce nephrotoxicity will provide better strategies to manage the kidney side effects without compromising their antineoplastic benefit.

## Figures and Tables

**Table 1:** Function of constitutively expressed Vascular Endothelial Growth Factor (VEGF) and VEGF receptors in the normal kidney.

Embryonic stage and development
• Endothelial cell differentiation, migration, and maturation during nephron formation (metanephric stage);
• Induction of fenestrations, transcellular gaps, caveolae, and inter-endothelial gaps.
Adult stage, developed kidney
• Vascular permeability/regulation of glomerular permeability;
• Regulation of slit diaphragm by upregulation of podocin and its interaction with CD2AP;
• Protection of renal tubular cells;
• Maintenance of basement membrane composition;
• Calcium homeostasis and podocyte survival;
• Mediation of endothelium dependent vasodilation;
• Remodeling of interstitial matrix.

VEGF, vascular endothelial growth factor; CD2AP, CD2-associated protein.

## References

[1] Capasso A., Benigni A., Capitanio U., Danesh F.R., Di Marzo V., Gesualdo L., Grandaliano G., Jaimes E.A., Malyszko J., Perazella M.A. (2019). International conference on onco-nephrology participants. Summary of the international conference on onco-nephrology: An emerging field in medicine. Kidney Int..

[2] Nussbaum E.Z., Perazella M.A. (2018). Update on the nephrotoxicity of novel anticancer agents. Clin. Nephrol..

[3] Sury K., Perazella M.A. (2019). The nephrotoxicity of new immunotherapies. Expert Rev. Clin. Pharmacol..

[4] Abbas A., Mirza M.M., Ganti A.K., Tendulkar K. (2015). Renal toxicities of targeted therapy. Targ. Oncol..

[5] Estrada C.C., Maldonado A., Mallipattu S.K. (2019). Therapeutic inhibition of VEGF signaling and associated nephrotoxicities. J. Am. Soc. Nephrol..

[6] Folkman J. (1971). Tumor angiogenesis: Therapeutic implications. N. Engl. J. Med..

[7] Larcher F., Robles A.I., Duran H., Murillas R., Quintanilla M., Cano A., Conti C.J., Jorcano J.L. (1996). Up-regulation of vascular endothelial growth factor/vascular permeability factor in mouse skin carcinogenesis correlates with malignant progression state and activated H-ras expression levels. Cancer Res..

[8] Ferrara N., Gerber H.P., LeCouter J. (2003). The biology of VEGF and its receptors. Nat. Med..

[9] Miettinen M., Rikala M.S., Rys J., Lasota J., Wang Z.F. (2012). Vascular endothelial growth factor receptor 2 as a marker for malignant vascular tumors and mesothelioma: An immunohistochemical study of 262 vascular endothelial and 1640 nonvascular tumors. Am. J. Surg. Pathol..

[10] Sugimoto H., Hamano Y., Charytan D., Cosgrove D., Kieran M., Sudhakar A., Kalluri L. (2003). Neutralization of circulating vascular endothelial growth factor [VEGF] by anti-VEGF antibodies and soluble VEGF receptor 1 (sFlt-1) induces proteinuria. J. Biol. Chem..

[11] Gurevich F., Perazzella M.A. (2009). Renal effects of anti-angiogenesis therapy: Update for the internist. Am. J. Med..

[12] Holash J., Davis S., Papadopoulos N., Croll S.D., Ho L., Russell M., Boland P., Leidich R., Hylton D., Burova E. (2002). VEGF-Trap: A VEGF blocker with potent antitumor effects. Proc. Natl. Acad. Sci. USA.

[13] Hamnvik O.P., Choueiri T.K., Turchin A., McKay R.R., Goyal L., Davis M., Kaymakcalan M.D., Williams J.S. (2015). Clinical risk factors for the development of hypertension in patients treated with inhibitors of the VEGF signaling pathway. Cancer.

[14] Izzedine H., Massard C., Spano J.P., Goldwasser F., Khayat D., Soria J.C. (2009). VEGF signaling inhibition-induced proteinuria: Mechanisms significance and management. Eur. J. Cancer.

[15] Qi W.X., Shen Z., Tang L.N., Yao Y. (2014). Risk of hypertension in cancer patients treated with aflibercept: A systematic review and metaanalysis. Clin. Drug Investig..

[16] Hood J.D., Meininger C.J., Ziche M., Granger H.J. (1998). VEGF upregulates eNOS message, protein, and NO production in human endothelial cells. Am. J. Physiol..

[17] Zou A.P., Cowley A.W. (1999). Role of nitric oxide in the control of renal function and salt sensitivity. Curr. Hypertens. Rep..

[18] Robinson E.S., Khankin E.V., Karumanchi S.A., Humphreys B.D. (2010). Hypertension induced by vascular endothelial growth factor signaling pathway inhibition: Mechanisms and potential use as a biomarker. Semin. Nephrol..

[19] Gonzalez-Pacheco F.R., Deudero J.J., Castellanos M.C., Castilla M.A., Alvarez-Arroyo M.V., Yague S., Caramelo C. (2006). Mechanisms of endothelial response to oxidative aggression: Protective role of autologous VEGF and induction of VEGFR2 by H2O2. Am. J. Physiol. Heart Circ. Physiol..

[20] Kanbayashi Y., Ishikawa T., Tabuchi Y., Sakaguchi K., Ouchi Y., Otsuji E., Takayama K., Taguchi T. (2020). Predictive factors for the development of proteinuria in cancer patients treated with bevacizumab, ramucirumab, and aflibercept: A single-institution retrospective analysis. Sci. Rep..

[21] Kamba T., Tam B.Y., Hashizume H., Haskell A., Sennino B., Mancuso M.R., Norberg S.M., O’Brien S.M., Davis R.B., Gowen L.C. (2006). VEGF-dependent plasticity of fenestrated capillaries in the normal adult microvasculature. Am. J. Physiol. Heart Circ. Physiol..

[22] Izzedine H., Escudier B., Lhomme C., Pautier P., Rouvier P., Gueutin V., Baumelou A., Derosa L., Bahleda R., Hollebecque A. (2014). Kidney diseases associated with anti-vascular endothelial growth factor (VEGF) An 8-year observational study at a single center. Medicine.

[23] Izzedine H., Mangier M., Ory V., Zhang S.Y., Sendeyo K., Bouachi K., Audard V., Pe’choux C., Soria J.C., Massard C. (2014). Expression patterns of RelA and c-mip are associated with different glomerular diseases following anti-VEGF therapy. Kidney Int..

[24] Izzedine H., Isnard-Bagnis C., Launay-Vacher V., Mercadal L., Tostivint I., Rixe O., Brocheriou I., Bourry E., Karie S., Saeb S. (2006). Gemcitabine induced thrombotic microangiopathy: A systematic review. Nephrol. Dial. Transplant..

[25] Takase O., Marumo T., Imai N., Hirahashi J., Takayanagi A., Hishikawa K., Hayashi M., Shimizu N., Fujita T., Saruta T. (2005). NF-kappaB-dependent increase in intrarenal angiotensin II induced by proteinuria. Kidney Int..

[26] Maitland M.L., Bakris G.L., Black H.R., Chen H.X., Durand J.B., Elliott W.J., Ivy S.P., Leier C.V., Lindenfeld J., Liu G. (2010). Cardiovascular toxicities panel, convened by the angiogenesis task force of the national cancer institute investigational drug steering committee: Initial assessment, surveillance, and management of blood pressure in patients receiving vascular endothelial growth factor signaling pathway inhibitors. J. Natl. Cancer Inst..

[27] Grenon N.N. (2013). Managing toxicities associated with antiangiogenic biologic agents in combination with chemotherapy for metastatic colorectal cancer. Clin. J. Oncol. Nurs..

[28] Calizo R.C., Bhattacharya S., Coen van Hasselt J.G. (2019). Disruption of podocyte cytoskeletal biomechanics by dasatinib leads to nephrotoxicity. Nat. Commun..

[29] Demetri G.D., Russo L.O., Mac Pherson I.R., Wang D., Morgan J.A., Brunton V.G., Paliwal P., Agrawal S., Voi M., Evans T.R.J. (2009). Phase I dose escalation and farmacokinetic study of dasatinib in patients with advanced solid tumors. Clin. Cancer Res..

[30] Hirano T., Hashimoto M., Korogi Y., Tsuji T., Miyanaka K., Yamasaki H., Tsuda H. (2016). Dasatinib-induced nephrotic syndrome. Leuk. Lymphoma.

[31] Ruebner R.L., Copelovitch L., Evageliou N.F., Denburg M.R., Belasco J.B., Kaplan B.S. (2014). Nephrotic syndrome associated with tyrosine kinase inhibitors for pediatric malignancy: Case series and review of the literature. Pediatr. Nephrol..

[32] Lim Y.T., Kim Y.J., Park Y.H., Hah J.O., Lee J.M. (2016). A case of dasatinib-induced nephrotic syndrome in a child with Philadelphia chromosome positive acute lymphoblastic leukemia. Yonsei Med. J..

[33] Chow C., Abraham S., Arceci R.J., Newburger P.E. Dasatinib induced chylothorax and nephrotic syndrome in a pediatric patient. Pediatric Blood and Cancer, Proceedings of the American Society of Hematology/Oncology (ASPHO), Phoenix, AZ, USA, 6–9 May 2015.

[34] Ochiai S., Sato Y., Minakawa A., Fukuda A., Fujimoto S. (2019). Dasatinib-induced nephrotic syndrome in a patient with chronic myelogenous leukemia: A case report BMC. Nephrology.

[35] Wallace E., Lyndon W., Chumley P., Jaimes E.A., Fatima H. (2013). Dasatinib-induced nephrotic-range proteinuria. Am. J. Kidney Dis..

[36] De Luca M.L., Carmosino I., Stefanizzi C., Campanelli M., De Angelis F., Cesini L., Latagliata R., Alimena G. (2016). Nephrotic proteinuria developed under dasatinib treatment in a patient with chronic myeloid leukemia: A case report and review of the literature. Ann. Hematol. Oncol..

[37] Koinuma K., Sakairi T., Watanabe Y., IIzuka A., Watanabe M., Hamatani H., Nakasatomi M., Ishizaki T., Ikeuchi. H., Kaneko Y. (2020). A Case of long-term dasatinib-induced proteinuria and glomerular injury. CEN Case Rep..

[38] Jhaveri K.D., Wanchoo R., Sakhiya V., Ross D.W., Fishbane S. (2017). Adverse renal effects of novel molecular oncologic targeted therapies: A narrative review. Kidney Int. Rep..

[39] Muller-Hansma A.H.G., van der Lugt J., Zwaan C.M. (2017). Nephrotic syndrome under treatment with dasatinib: Be aware of a possible adverse drug reaction. Neth. J. Med..

[40] Liang W., Kujawski M., Wu J., Lu J., Herrmann A., Loera S., Yen Y., Lee F., Yu H., Wen W. (2010). Antitumor activity of targeting SRC kinases in endothelial and myeloid cell compartments of the tumor microenvironment. Clin. Cancer Res..

[41] Advani A. (2014). Vascular endothelial growth factor and the kidney: Something of the marvellous. Curr. Opin. Nephrol. Hypertens..

[42] Yang N., Higuchi O., Ohashi K., Nagata K., Wada A., Kangawa K., Nishida E., Mizuno K. (1998). Cofilin phosphorylation by LIM-kinase 1 and its role in Rac-mediated actin reorganization. Nature.

[43] Falkenberg C.V., Azeloglu E.U., Stothers M., Deerinck T.J., Chen Y., He J.C., Ellisman M.H., Hone J.C., Iyengar R., Loew L.M. (2017). Fragility of foot process morphology in kidney podocytes arises from chaotic spatial propagation of cytoskeletal instability. PLoS Comput. Biol..

[44] Embry A.E., Liu Z., Henderson J.M., Byfield F.J., Liu L., Yoon J., Wu Z., Cruz K., Moradi S., Gillombardo C.B. (2018). Similar biophysical abnormalities in glomeruli and podocytes from two distinct models. J. Am. Soc. Nephrol..

[45] Perazella M.A., Shirali A.C. (2020). Immune checkpoint inhibitor nephrotoxicity: What do we know and what should we do?. Kidney Int..

[46] Anari F., Ramamurthy C., Zibelman M. (2018). Impact of tumor microenvironment composition on therapeutic responses and clinical outcomes in cancer. Future Oncol..

[47] Yu Y., Cui J. (2018). Present and future of cancer immunotherapy: A tumor microenvironmental perspective. Oncol. Lett..

[48] Iwai Y., Hamanishi J., Chamoto K., Honjo T. (2017). Cancer immunotherapies targeting the PD-1 signaling pathway. J. Biomed. Sci..

[49] Luke J.J., Ott P.A. (2015). PD-1 pathway inhibitors: The next generation of immunotherapy for advanced melanoma. Oncotarget.

[50] Pico de Coaña Y., Choudhury A., Kiessling R. (2015). Checkpoint blockade for cancer therapy: Revitalizing a suppressed immune system. Trends Mol. Med..

[51] Wei S.C., Duffy C.R., Allison J.P. (2018). Fundamental mechanisms of immune checkpoint blockade therapy. Cancer Discov..

[52] Postow M.A., Sidlow R., Hellmann M.D. (2018). Immune-related adverse events associated with immune checkpoint blockade. N. Engl. J. Med..

[53] Wang P.F., Chen Y., Song S.Y., Wang T.J., Ji W.J., Li S.W., Liu N., Yan C.X. (2017). Immune-related adverse events associated with anti-PD-1/PD-L1 treatment for malignancies: A meta-analysis. Front. Pharmacol..

[54] Cortazar F.B., Marrone K.A., Troxell M.L., Ralto K.M., Hoenig M.P., Brahmer J.R., Le D.T., Lipson E.J., Glezerman I.G., Wolchok J. (2016). Clinicopathological features of acute kidney injury associated with immune checkpoint inhibitors. Kidney Int..

[55] Haslam A., Prasad V. (2019). Estimation of the percentage of US patients with cancer who are eligible for and respond to checkpoint inhibitor immunotherapy drugs. JAMA Netw. Open.

[56] Bavi P., Kiehl R., Adeyi O., Mete O., Avila-Casado C., Sharifzad H., Joshua A., Butany J., Roehrl M.H. Immune-related adverse events (irAEs) following CTLA-4 and PD-1/PD-L1 blockade in advanced melanoma: A comprehensive rapid autopsy study. Proceedings of the Annual Meeting of the United States and Canadian Academy of Pathology.

[57] Robert C., Long G.V., Brady B., Dutriaux C., Maio M., Mortier L., Hassel J.C., Rutkowski P., McNeil C., Kalinka-Warzocha E. (2015). Nivolumab is previously untreated melanoma without BRAF mutation. N. Engl. J. Med..

[58] Shirali A.C., Perazella M.A., Gettinger S. (2016). Association of acute interstitial nephritis with programmed cell death 1 inhibitor therapy in lung cancer patients. Am. J. Kidney Dis..

[59] Belliere J., Meyer N., Mazieres J., Ollier S., Boulinguez S., Delas A., Ribes D., Faguer S. (2016). Acute interstitial nephritis related to immune checkpoint inhibitors. Br. J. Cancer.

[60] Murakami N., Borges T.J., Yamashita M., Riella L.V. (2016). Severe acute interstitial nephritis after combination immune-checkpoint inhibitor therapy for metastatic melanoma. Clin. Kidney J..

[61] Escandon J., Peacock S., Trabolsi A., Thomas D.B., Layka A., Lutzky J. (2017). Interstitial nephritis in melanoma patients secondary to PD-1 checkpoint inhibitor. J. Immunother. Cancer.

[62] Koda R., Watanabe H., Tsuchida M., Iino N., Suzuki K., Hasegawa G., Imai N., Narita I. (2018). Immune checkpoint inhibitor (nivolumab)-associated kidney injury and the importance of recognizing concomitant medications known to cause acute tubulointerstitial nephritis: A case report. BMC Nephrol..

[63] Bajwa R., Cheema A., Khan T., Amirpour A., Paul A., Chaughtai S., Patel S., Patel T., Bramson J., Gupta V. (2019). Adverse effects of immune checkpoint inhibitors (programmed death-1 inhibitors and cytotoxic T-limphocyte- associated protein-4 inhibitors): Result of retrospective study. J. Clin. Med. Res..

[64] Mamlouk O., Selamet U., Machado S., Abdelrahim M., Glass W.S., Tchakarov A., Gaber L., Lahoti A., Workeneh B., Chen S. (2019). Nephrotoxicity of immune checkpoint inhibitors beyond tubulointerstitial nephritis: Single center experience. J. Immuno. Ther. Cancer.

[65] Okawa S., Fujiwara K., Shimonishi A., Matsuura H., Ozeki T., Nishimura J., Kayatani H., Minami D., Shinno Y., Sato K. (2020). Rapidly progressive acute kidney injury associated with nivolumab treatment. Case Rep. Oncol..

[66] Cortazar F.B., Kibbelaar Z.A., Glezerman I.G., Abudayyeh A., Mamlouk O., Motwani S.S., Murakami N., Herrmann S.M., Manohar S., Shirali A.C. (2020). Clinical features and outcomes of immune checkpoint inhibitor–associated AKI: A multicenter study. J. Am. Soc. Nephrol..

[67] Lin J., Schiff M., Salvatore S., Shoustari A., Glezerman I. Membranous nephropathy related to the checkpoint inhibitor nivolumab. Proceedings of the American Society of Nephrology Kidney Week.

[68] Daanen R.A., Maas R.J.H., Koornstra R.H.T., Steenbergen E.J., van Herpen C.M.L., Willemsen A.E.C.A.B. (2017). Nivolumab-associated nephrotic syndrome in a patient with renal cell carcinoma: A case report. J. Immunother..

[69] Ray A., Ghosh S., Ghosh M., Yarlagadda S. Nivolumab induced renal failure with collapsing focal segmental glomerulosclerosis (FSGS). Proceedings of the American Society of Nephrology Kidney Week.

[70] Crutz-Whitley J., Giehl N., Jen K., Young B.l. (2020). Membranoproliferative glomerulonephritis associated with nivolumab therapy. Case Rep. Nephrol..

[71] Jung K., Zeng X., Bilusic M. (2016). Nivolumab-associated acute glomerulonephritis; a case report and literature review. BMC Nephrol..

[72] Kishi S., Minato M., Saigo A., Murakami N., Tamaki M., Matsuura M., Murakami T., Nagai K., Abe H., Nishioka Y. (2018). IgA nephropathy after nivolumab therapy for postoperative recurrence of squamous cell carcinoma. Intern. Med..

[73] Francisco L.M., Sage P.T., Sharpe A.H. (2010). The PD-1 pathway in tolerance and autoimmunity. Immunol. Rev..

[74] Schoop R., Wahl P., Le Hir M., Heemann U., Wang M., Wüthrich R.P. (2004). Suppressed T-cell activation by IFNgamma-induced expression of PD-L1 on renal tubular epithelial cells. Nephrol. Dial. Transplant..

[75] Kuchroo V.K., Ohashi P.S., Sartor R.B., Vinuesa C.G. (2012). Dysregulation of immune homeostasis in autoimmune diseases. Nat. Med..

[76] Perazella M.A. (2020). Kidney biopsy should be performed to document the cause of immune checkpoint inhibitor–associated acute kidney injury: Commentary. Kidney360.

[77] Gutgarts V., Glezerman I.G. (2020). Kidney biopsy should be performed to document the cause of immune checkpoint inhibitor–associated acute kidney injury: CON. Kidney360.

[78] Eijgelsheim M., Sprangers B. (2020). Kidney biopsy should be performed to document the cause of immune checkpoint inhibitor–associated acute kidney injury: PRO. Kidney360.

[79] Moledina D.G., Wilson F.P., Pober J.S., Perazella M.A., Singh N., Luciano R.L., Obeid W., Lin H., Kuperman M., Moeckel G.W. (2019). Urine TNF-a and IL-9 for clinical diagnosis of acute interstitial nephritis. JCI Insight.

[80] Yekeduz E., Dursun B., Aydin G.C., Yazgan S.C., Öztürk H.H., Azap A., Utkan G., Ürün Y. (2020). Clinical course of COVID-19 infection in elderly patient with melanoma on nivolumab. J. Oncol. Pharm. Pract..

[81] Cosmai L., Porta C., Perazella M., Launay-Vacher V., Rosner M.H., Kenar J., Floris M., Pani A., Teuma C., Szczylik C. (2018). Opening an onconephrology clinic: Recommendations and basic requirements. Nephrol. Dial. Transplant..

[82] Izzedine H., Perazella M.A. (2017). Anticancer drug-induced acute kidney injury. Kidney Int. Rep..

[83] Hanna R.M., Barsoum M., Arman F., Selamet U., Hasnain H., Kurtz I. (2019). Nephrotoxicity induced by intravitreal vascular endothelial growth factor inhibitors: Emerging evidence. Kidney Int..

[84] De Jesus-Gonzalez N., Robinson E., Moslehi J., Humphreys B.D. (2012). Management of antiangiogenic therapy-induced hypertension. Hypertension.

[85] Sprangers B., Abudayyeh A., Latcha S., Perazella M.A., Jhaveri K.D. (2020). How to determine kidney function in cancer patients?. Eur. J. Cancer.

[86] Janowitz T., Williams E.H., Marshall A., Ainsworth N., Thomas P.B., Sammut S.J., Shepherd S., White J., Mark P.B., Lynch A.G. (2017). New model for estimating glomerular filtration rate in patients with cancer. J. Clin. Oncol..

[87] Tool to Estimate Glomerular Filtration. http://tavarelab.cruk.cam.ac.UK/JanowitzWulliamsGFR.

[88] Okamoto S., Ureshino H., Kawaguchi A., Miyazono M., Ikeda Y., Kimura S. (2020). Assessment of estimated glomerular filtration rate in patients with chronic myeloid leukemia following discontinuation of tyrosine kinase inhibitors. Int. J. Hematol..

[89] Sirolli V., Pieroni L., Di Liberato L., Urbani A., Bonomini M. (2019). Urinary peptidomic biomarkers in kidney diseases. Int. J. Mol. Sci..

